# Being Born Small for Gestational Age or With Intrauterine Growth Restriction Impairs Adult Anthropometric Development

**DOI:** 10.1111/apa.70036

**Published:** 2025-02-25

**Authors:** Achim Fieß, Sandra Gißler, Dirk Wackernagel, Julia Winter, Norbert Pfeiffer, Alexander K. Schuster, Eva Mildenberger, Alica Hartmann

**Affiliations:** ^1^ Department of Ophthalmology University Medical Center of the Johannes Gutenberg University Mainz Mainz Germany; ^2^ Division of Neonatology, Department of Pediatrics University Medical Center of the Johannes Gutenberg University Mainz Mainz Germany

Previous studies have shown that fetal growth restriction leads to reduced body proportions, with individuals born small for gestational age (SGA) being shorter and having less body weight [1, 2]. However, these studies have not consistently distinguished between individuals born SGA and those with intrauterine growth restriction (IUGR). SGA is typically defined by a birth weight (BW) below the 10th percentile for gestational age, without accounting for in utero growth patterns. In contrast, IUGR refers to impaired fetal growth due to placental dysfunction, for example, malnutrition or other factors during gestation, irrespective of BW percentile [3]. Providing targeted nutritional guidelines for these vulnerable groups could be essential to mitigate deficits in anthropometric outcomes [4]. Thus, it is highly clinically relevant to clearly differentiate whether these effects are specific to the SGA group, the IUGR group, or both. This distinction allows for tailored interventions that address the unique needs and risks associated with each condition, enhancing the precision and effectiveness of support for long‐term health and growth.

The Gutenberg Prematurity Study (GPS) is a retrospective cohort study in Germany, complemented by a prospective examination. Participants, selected through an algorithm, include adults aged 18–52 years who were born preterm or at term, as described previously [5]. They completed structured interviews and underwent comprehensive examinations. Additionally, detailed perinatal parameters, including birth weight, were collected during participant interviews. To ensure accuracy, these data were validated with medical records. To analyse the participants, they were grouped (*n* = 105) into three groups (Group 1: controls [*n* = 35], Group 2: IUGR [n = 35], Group 3: SGA without IUGR [n = 35]), and the groups were matched by age (date at study examination), sex and gestational age. SGA was defined by a birth weight below the 10th percentile for gestational age, while IUGR status was determined solely based on documentation extracted from the patients' medical records. During the study period in Germany, the diagnosis of IUGR was typically based on clinical indicators such as falling fetal growth trajectories observed during pregnancy, birth weight below the 10th percentile with evidence of placental insufficiency, or abnormal Doppler ultrasound findings indicating compromised fetal circulation. The control group had no evidence of SGA or IUGR. A linear regression model was used with anthropometric parameters (adult body height, adult body weight, adult head circumference and adult body mass index [BMI]) as dependent variables and with adjustment for age (years), sex (female) and gestational age (weeks). Additionally, a sensitivity analysis was conducted to account for maternal anthropometric characteristics (height, weight and BMI) as potential confounders. However, it should be noted that maternal data were available for only approximately 50% of the participants, and head circumference measurements for mothers were not collected. Paternal anthropometric data were excluded due to lower availability. All statistical analyses were conducted using R version 4.3.3.

In this analysis, 105 participants were included (age 26.1 ± 5.6 years, 57 women). BW percentiles differed between the groups as expected (BW percentiles: control: 41.97 ± 25.98; IUGR: 6.54 ± 11.67; SGA: 3.77 ± 2.50), while age, sex and gestational age were well balanced between the groups, as a result of the matching. Descriptive distribution of anthropometric parameters in the different groups is presented in Figure 1. In the multivariable analysis, individuals born SGA or IUGR had significantly lower adult body height compared to controls (SGA: *B* = −4.69, 95% CI = −7.74 to −1.64, *p* = 0.003; IUGR: *B* = −4.51, 95% CI = −7.53 to −1.49, *p* = 0.004). SGA was also associated with lower adult body weight (*B* = −9.39, 95% CI = −15.57 to −3.21, *p* = 0.003). Adult head circumference was smaller in both the SGA group (*B* = −0.92, 95% CI = −1.81 to −0.04, *p* = 0.04) and the IUGR group (*B* = −1.04, 95% CI = −1.91 to −0.17, *p* = 0.02) compared to controls. No significant differences in adult BMI were observed between groups. In a sensitivity analysis adjusting for maternal anthropometric parameters (height, weight and BMI), the association between being born IUGR and reduced adult body height remained significant (*B* = −5.37, CI = −9.64 to −1.10, *p* = 0.01), while the association with SGA without IUGR was no longer significant. The association between adult body weight and SGA remained consistent (*B* = −9.06, CI = −16.59 to −1.53, *p* = 0.02), and a new association with IUGR was observed (*B* = −9.59, CI = −16.90 to −2.28, *p* = 0.01). No significant associations with adult BMI were observed for either SGA or IUGR. Additionally, maternal weight and BMI were independently associated with the anthropometric outcomes of the offspring, whereas maternal height did not.

**FIGURE 1:**
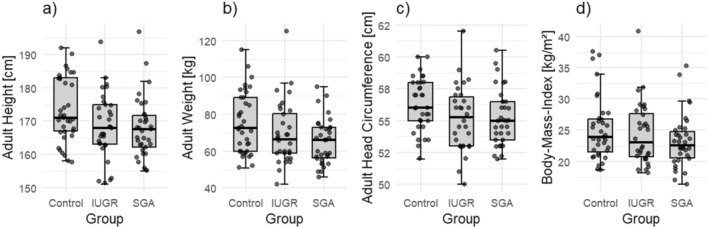
Boxplots showing anthropometric measures in adulthood grouped by control, individuals with intrauterine growth restriction and individuals being born small for gestational age: (a) body height [cm], (b) body weight [kg], (c) head circumference [cm] and (d) body‐mass‐index [kg/m^2^]. IUGR, intrauterine growth restriction; SGA, small for gestational age.

In this matched analysis, we provide new data that both being born SGA (without IUGR) and having a history of IUGR independently result in reduced adult body height and smaller adult head circumference compared to the control group. Additionally, an association was found between being born SGA and having a lower adult body weight. Sensitivity analyses adjusting for maternal anthropometric parameters revealed differences compared to the main analysis. While the association between IUGR and reduced adult height remained robust, the association with SGA was no longer significant. For adult body weight, the association with SGA remained consistent, and a new association with IUGR emerged. These findings highlight the importance of considering additional factors that may influence the observed relationships and emphasise the need for further research to disentangle these complex interactions. One limitation of this study is that a large proportion of participants with IUGR (83%) were also SGA, which may reduce the isolated effect of IUGR in our study; therefore, we included this factor in the multivariable model. Another limitation is the relatively small number of cases in our cohort. This limited sample size may affect the precision of our estimates and restrict the generalisability of our findings. Future studies with larger cohorts are needed to validate these findings and provide a more comprehensive understanding of the distinct impacts of SGA and IUGR on adult anthropometry. Moreover, one limitation is the widespread lack of awareness among colleagues regarding the distinction between SGA and IUGR, which may lead to inconsistencies in definitions and interpretations. Additionally, the lack of standardisation of IUGR criteria across the study period introduces potential variability, which may limit the generalisability and comparability of our findings. Future studies should employ prospective designs with predefined, standardised diagnostic criteria to ensure greater consistency and reliability.

Overall, the results indicate that both being born SGA (without IUGR) and IUGR negatively influence adult anthropometric development; however, there is a need for future research employing prospective studies with predefined SGA and IUGR criteria.

## Author Contributions

**Achim Fieß:** conceptualization, writing – original draft, validation, formal analysis, writing – review and editing. **Sandra Gißler:** writing – original draft, validation, formal analysis, writing – review and editing. **Dirk Wackernagel:** validation, writing – review and editing, formal analysis. **Julia Winter:** validation, writing – review and editing, formal analysis. **Norbert Pfeiffer:** validation, writing – review and editing, formal analysis. **Alexander K. Schuster:** validation, writing – review and editing, formal analysis, conceptualization. **Eva Mildenberger:** validation, writing – review and editing, formal analysis. **Alica Hartmann:** validation, writing – review and editing, formal analysis, writing – original draft, visualization.

## Ethics Statement

All participants provided written informed consent in accordance with Good Clinical Practice, Good Epidemiological Practice and the Declaration of Helsinki. The study protocol was approved by the Medical Chamber of Rhineland‐Palatinate's ethics committee (reference no. 2019–14 161; original vote: 29.05.2019, latest update: 02.04.2020).

## Consent

The authors have nothing to report.

## Conflicts of Interest

Pfeiffer N receives financial support and grants from Novartis, Ivantis, Santen, Thea, Boehringer Ingelheim Deutschland GmbH & Co. KG, Alcon and Sanoculis. Schuster AK receives research support from Allergan, Bayer, Heidelberg Engineering, PlusOptix and Norvartis. Fieß A, Gißler S, Wackernagel D, Winter J, Mildenberger E and Hartmann A: none.

## References

[1] T. Meas , S. Deghmoun , P. Armoogum , C. Alberti , and C. Levy‐Marchal , “Consequences of Being Born Small for Gestational Age on Body Composition: An 8‐Year Follow‐Up Study,” Journal of Clinical Endocrinology and Metabolism 93 (2008): 3804–3809. 18628518 10.1210/jc.2008-0488 PMC2579646

[2] A. Suhag , A. Rerkasem , K. Kulprachakarn , et al., “Long‐Term Health Associated With Small and Large for Gestational Age Births Among Young Thai Adults,” Children 9 (2022): 779. 35740716 10.3390/children9060779 PMC9221860

[3] D. Sharma , S. Shastri , and P. Sharma , “Intrauterine Growth Restriction: Antenatal and Postnatal Aspects,” Clinical Medicine Insights Pediatrics 10 (2016): 67–83. 27441006 10.4137/CMPed.S40070 PMC4946587

[4] F. Haschke , C. Binder , M. Huber‐Dangl , and N. Haiden , “Early‐Life Nutrition, Growth Trajectories, and Long‐Term Outcome,” Nestle Nutrition Institute Workshop Series 90 (2019): 107–120. 30865980 10.1159/000490299

[5] A. Fieß , K. Dautzenberg , S. Gißler , et al., “Prevalence of Strabismus and Risk Factors in Adults Born Preterm With and Without Retinopathy of Prematurity: Results From the Gutenberg Prematurity Eye Study,” British Journal of Ophthalmology 108 (2024): 1590–1597. 38503479 10.1136/bjo-2023-324698 PMC11503079

